# A Four-Decade Bibliometric Analysis on the Genera, *Asphodelus* L. and *Asphodeline* Rchb., Belonging to the Family Asphodelaceae: Research Trends and Knowledge Gaps

**DOI:** 10.3390/plants15101421

**Published:** 2026-05-07

**Authors:** Giuseppe Natale Basile, Claudio Calia, Sajid Safeer, Claudia Ruta, Giuseppe De Mastro

**Affiliations:** Department of Soil, Food and Plant Sciences, University of Bari Aldo Moro, 70126 Bari, Italy; giuseppe.basile@uniba.it (G.N.B.); claudio.calia@uniba.it (C.C.); sajid.safeer@uniba.it (S.S.); giuseppe.demastro@uniba.it (G.D.M.)

**Keywords:** wild plants, plant extract, MAPs, Mediterranean flora, phytochemistry, PRISMA, VOSviewer

## Abstract

The family Asphodelaceae, particularly the genera *Asphodelus* L. and *Asphodeline* Rchb., includes Mediterranean wild plants of ecological, agronomic, and phytochemical importance. Yet, their applied scientific studies remain fragmented and lack synthesis. This study provides the first bibliometric analysis of applied agronomic, ecological, and phytochemical research on these genera, integrating a transparent and reproducible framework. Publications indexed in the Scopus database from 1987 to 2026 were systematically screened. A sensitivity analysis of the search string progressively reduced the corpus from 1797 taxonomy-only records to 149 after applying intervention, outcomes, and exclusion criteria, of which 64 studies met the final inclusion criteria (43% inclusion rate). Bibliometric analysis was performed using Bibliometrix and VOSviewer. The final dataset spans 57 sources, with 265 contributing authors, an average of 4.95 authors per paper, and an international collaboration rate of 22.15%, highlighting the interdisciplinary nature of the field. Scientific production shows a gradual increase over time, with peaks after 2019, although citation impact remains uneven (mean 15.23 citations per document) and concentrated in a few highly cited studies. Research is geographically clustered along a Mediterranean–South Asian axis, with Italy emerging as the main hub of citation impact and collaboration. Keyword analysis identifies four main thematic clusters, revealing a progressive shift from early ecological and weed–crop interaction studies toward phytochemistry, plant extracts, flavonoids, and antioxidant activity. Despite this growth, substantial gaps persist in agronomy, domestication, and large-scale cultivation, limiting the translation of these species into viable crops. The present analysis is therefore motivated by the need to clarify the current level of scientific knowledge on these species and to assess their potential for future domestication. In this context, identifying research gaps is essential not only for guiding interdisciplinary studies but also for supporting the development of value chains and processing pathways aimed at the sustainable valorisation of these underexplored Mediterranean species.

## 1. Introduction

The genera *Asphodelus* and *Asphodeline* (family Asphodelaceae, order Asparagales) comprise annual and perennial geophytic species widely distributed across the Mediterranean basin and adjacent arid and semiarid regions. These taxa are characterized by well-developed underground reserve organs, including fleshy roots and short rhizomes, which support seasonal regrowth and confer high resilience to environmental stress. Such traits allow these species to thrive in marginal, disturbed, and nutrient-poor habitats, where they often form stable and persistent populations [1,2].

Morphologically, species of both genera typically develop erect flowering scapes bearing actinomorphic flowers with six free tepals and prominent stamens, favoring entomophilous pollination. Reproduction occurs either sexually through seeds or vegetatively via rhizome multiplication in perennial taxa, a dual strategy that enhances dispersal capacity and ecological adaptability [3,4,5]. Their life cycles are closely aligned with Mediterranean climatic conditions, with spring flowering followed by summer dormancy, reflecting an effective adaptation to seasonal water limitation [6,7].

Phylogenetically, *Asphodelus* and *Asphodeline* share a common evolutionary origin within the Asphodelaceae, as confirmed by analyses of plastid and nuclear DNA [8,9]. Despite overall morphological similarity, interspecific variation in floral traits, root architecture, and ecological preferences has driven diversification, with speciation often associated with geographical isolation and local environmental gradients [10]. Several species, including *Asphodelus ramosus* L. [11,12,13], *Asphodelus albus* Mill. [14], *Asphodelus fistulosus* L. [15], *Asphodeline lutea* (L.) Rchb. [16,17], and *Asphodeline liburnica* (Scop.) Rchb. [18], are of particular ecological, ethnobotanical, and phytochemical relevance in the Mediterranean region, especially in Italy and the southern Apulia region [19,20].

Beyond their native range, some species, most notably *A. fistulosus* L., have successfully naturalized in other parts of the world, highlighting both their ecological plasticity and, in some contexts, their invasive potential [21,22]. Within Mediterranean agroecosystems, however, *Asphodelus* species play beneficial roles in soil stabilization, support of pollinator communities, and traditional agropastoral systems, where they have historically been used as fodder plants and soil enhancers [23].

The ecological success of these genera in disturbed environments has been linked to their life-history traits, notably their geophytic growth form and the persistence of underground storage organs. These traits confer high resistance to summer fires and enable rapid post-disturbance regeneration, thereby enabling the species to persist and expand under disturbance regimes that suppress less resilient competitors. Such characteristics explain the frequent association of these plants with repeatedly disturbed Mediterranean landscapes [24].

Over the last two decades, scientific interest in these genera has expanded markedly, particularly in relation to their phytochemical composition and biological activities. Numerous studies have identified bioactive secondary metabolites, including flavonoids, anthraquinones, phenolic acids, and saponins, using advanced analytical techniques such as HPLC, MS, and NMR [25,26]. These compounds have been associated with antioxidant, antimicrobial, anti-inflammatory, and cytotoxic properties, supporting the traditional medicinal use of *Asphodelus* and *Asphodeline* species [27,28,29,30].

Despite this growing body of the literature, most recent research remains largely focused on phytochemistry, while agronomic aspects are still underexplored. Studies addressing domestication strategies, cultivation requirements, breeding programs, and large-scale production systems are scarce or absent. This knowledge gap currently limits the translation of these wild species into economically viable crops. Given the increasing demand for sustainable and climate-resilient plant resources, a systematic evaluation of the existing research landscape is therefore needed [31,32,33,34,35].

In this context, a bibliometric approach offers a powerful tool to quantitatively assess scientific output, identify research trends, key contributors, and thematic evolution, and highlight critical gaps requiring future investigation [36]. The present study contributes to this need by providing the first comprehensive bibliometric review of research on *Asphodelus* and *Asphodeline*, considering plants belonging to these genera of potential practical interest and developing research on ecological, agronomic, and phytochemical aspects. By systematically mapping the literature, this work aims to evaluate whether existing knowledge is sufficient to support future domestication and cultivation strategies. Furthermore, identifying underexplored areas is essential for guiding interdisciplinary research and for defining possible value chains and processing pathways that could enable the sustainable valorisation of these wild species. This approach enables the analysis of the temporal evolution of research, the recognition of influential authors and institutional collaborations, and the identification of emerging trends [37]. Despite its growing adoption across numerous scientific disciplines, bibliometric analysis has not yet been applied to these Mediterranean wild plants belonging to the family of Asphodelaceae.

### Study Objectives and Scope

Building on these considerations, the present study aims to provide the first comprehensive bibliometric review of scientific research on *Asphodelus* and *Asphodeline*. The analysis focuses on publications indexed in the Scopus database over an extended temporal period, allowing for the examination of long-term research dynamics. Specifically, the objectives of this study are to:

i. assess the growth and distribution of scientific production;
ii. analyze citation patterns and research impact;
iii. identify leading institutions and collaboration networks;
iv. explore the thematic structure and evolution of research topics;
v. highlight critical knowledge gaps and future research directions.

By integrating ecological, agronomic, and phytochemical perspectives, this study seeks to provide a holistic understanding of the research landscape surrounding these genera. Ultimately, it aims to support the development of interdisciplinary strategies that promote the sustainable utilization and valorization of these underexplored plant resources.

## 2. Materials and Methods

Building on the need to systematically synthesize the fragmented body of knowledge identified in the Introduction, a bibliometric approach was adopted to quantitatively and qualitatively assess the scientific literature on the genera *Asphodelus* and *Asphodeline*. The methodological framework was designed to ensure transparency, reproducibility, and analytical rigor, following established protocols for bibliometric and systematic reviews [38,39]. The workflow consisted of four sequential steps: definition of the research strategy, application of inclusion and exclusion criteria, data extraction, and bibliometric and network analysis. In addition to the quantitative evaluation, a qualitative assessment was conducted during the screening process to identify the main thematic areas and research directions associated with these genera. This integrated approach ensured that the resulting dataset was both comprehensive and contextually aligned with the study objectives.

### 2.1. Data Source and Search Strategy

To ensure comprehensive coverage of the scientific literature, bibliographic data were retrieved from the Scopus database, which is widely recognized for its extensive indexing of peer-reviewed publications and its compatibility with advanced bibliometric tools. The search was conducted on 20 January 2026 and included documents published in English between 1987 and 2026. To systematically capture relevant records, the following Boolean search string was employed [40]:

TITLE-ABS-KEY(((“Asphodelus*” OR “Asphodeline*” OR asphodel* OR “*Asphodelus ramosus*” OR “*Asphodelus albus*” OR “*Asphodelus fistulosus*” OR “*Asphodeline lutea*” OR “*Asphodeline liburnica*” OR “*Asphodelus liburnicus*” OR “Asphodeline taurica” OR “*Asphodelus delphinensis*” OR “*Asphodelus pyrenaicus*” OR “*Asphodelus luteus*” OR “branched asphodel” OR “Mediterranean asphodel” OR “yellow asphodel”) AND (domesticat* OR propagat* OR “seed germination” OR agronom* OR cultivation OR “cultivation practice*” OR “agronomic practice*” OR field OR “field trial*” OR “open field” OR ecotype* OR characteri?ation OR “agronomic characteri?ation” OR “abiotic stress” OR drought OR “stress tolerance” OR habitat OR ecology OR soil OR “soil fertility” OR “soil properties” OR nutrient* OR nitrogen OR phosphorus OR potassium OR NPK OR compost OR manure OR “organic amendment*” OR micropropagation OR “in vitro” OR “tissue culture” OR callus OR embryo* OR extraction OR “solvent extraction” OR maceration OR soxhlet OR “ultrasound assisted” OR “microwave assisted” OR “plant extract*” OR phytochemical* OR “secondary metabolite*” OR chromatography OR GC-MS OR HPLC OR “LC-MS” OR “post-harvest”) AND (biomass OR “biomass production” OR yield OR productivity OR “yield components” OR growth OR “growth performance” OR “plant growth” OR “plant development” OR “vegetative growth” OR “root development” OR “shoot growth” OR phenology OR emergence OR “fresh weight” OR “dry weight” OR “dry matter” OR “survival rate” OR “field performance” OR “crop performance” OR “crop establishment” OR adaptability OR “extract yield” OR “chemical composition” OR “phytochemical composition” OR phenol* OR polyphenol* OR flavonoid* OR saponin*) AND NOT (aloe OR “aloe vera” OR kniphofia OR hemerocallis OR “day lily” OR bulbine OR human OR humans OR patient* OR clinical OR therapy OR therapeutic OR cancer OR tumor OR tumour OR disease OR medicine OR pharmacolog*))).

To assess the impact of the search string design on corpus composition, a sensitivity analysis was performed by progressively restricting the query (Table 1). A taxonomy-only search retrieved 1797 records, representing the theoretical upper bound of literature mentioning the target genera. The addition of the intervention and outcomes blocks (without the exclusion clause) reduced the corpus to 452 records (−75%), indicating that the majority of the broader literature on these genera consists of taxonomic, floristic, phylogenetic, or descriptive studies that do not address the applied agronomic, ecological, or phytochemical dimensions defined in the PICOL framework (Table 2). The application of the final exclusion (NOT) block further reduced the corpus to 149 records (−67% relative to the previous step, corresponding to approximately 8.3% of the taxonomy-only baseline). A qualitative inspection of the records excluded by the NOT block points out that they primarily comprised: (i) clinical and pharmacological studies on plant extracts; (ii) records retrieved through the wildcard “asphodel*” but referring to non-target taxa, most notably *Anemarrhena asphodeloides*; and (iii) studies on the excluded genera *Aloe*, *Kniphofia*, *Hemerocallis*, and *Bulbine*. This sensitivity analysis evidences that the final corpus reflects, by design, the applied research scope defined in the study objectives, rather than the totality of publications mentioning the target genera.

To ensure conceptual clarity and methodological consistency, the search query was structured around the five PICOL components: population, intervention, comparator, outcome, and location (Table 2). The search strategy was developed to capture studies addressing ecological, agronomic, botanical, and phytochemical aspects of selected target species and genera of the Asphodelaceae family. Both genus and species-level terms were included to maximize taxonomic coverage and minimize the risk of omission. Target species included *Asphodelus ramosus*, *A. albus*, *A. fistulosus*, *Asphodeline lutea*, *A. liburnica*, and related taxa characteristic of the Mediterranean region. The query integrated terms related to cultivation practices, propagation methods, stress responses, and phytochemical analysis, including extraction techniques and analytical methods such as GC-MS, HPLC, and LC-MS. In addition, keywords related to biomass production, growth performance, and metabolite composition were included to capture both agronomic and biochemical outcomes. At the same time, specific exclusion terms were applied to avoid retrieving studies related to non-target genera, such as *Aloe*, *Kniphofia,* or unrelated clinical contexts. This ensured that the final dataset remained focused on plant-based and applied research aspects relevant to the study objectives.

### 2.2. Data Extraction and Database Construction

Following the screening phase, relevant bibliographic information was extracted from each selected document. The metadata included title, authors, year of publication, journal source, document type, total citations, author keywords, index keywords, and author affiliations. These variables were selected to enable both descriptive and relational analysis of the scientific literature. The extracted data were organized into a structured database, which served as the basis for subsequent bibliometric analysis. To ensure data consistency and facilitate comparative assessments, each record was manually reviewed to verify taxonomic accuracy and to resolve any discrepancies in keyword classification. Additionally, geographic information associated with author affiliations was geocoded to enable spatial analysis of research distribution across regions, particularly within the Mediterranean basin, where *Asphodelus* and *Asphodeline* genera are predominantly studied. The inclusion of both bibliographic and keyword information allowed for the exploration of research productivity, collaboration patterns, and thematic evolution within the field [41,42].

### 2.3. Eligibility Criteria and Screening Process

**Figure 1:**
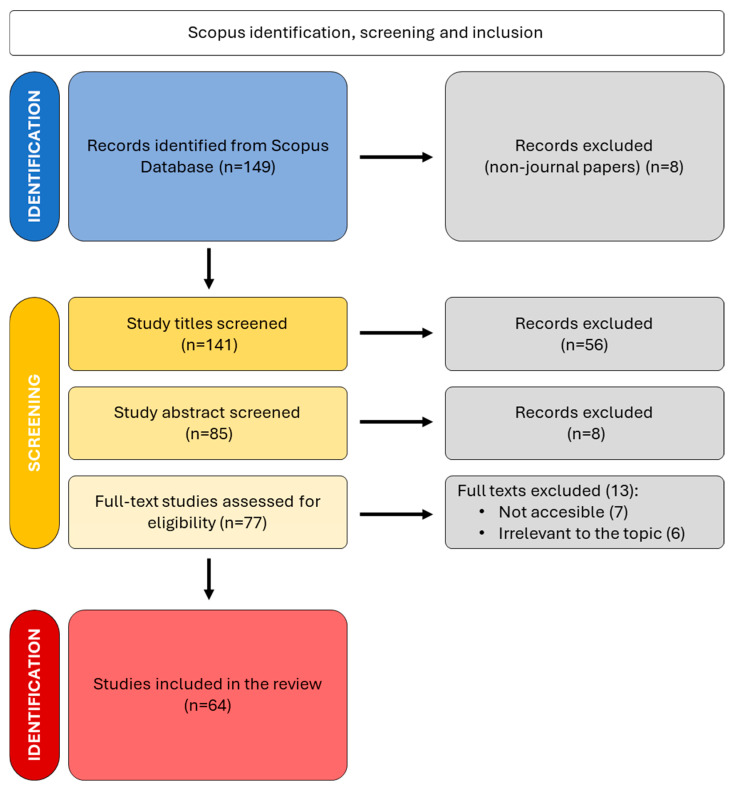
PRISMA flowchart depicting the article selection procedure (*n* represents the number of studies). Sequential phases of database identification, exclusion of non-journal papers, title screening, abstract screening, full-text eligibility assessment, exclusion of inaccessible or irrelevant studies, and final inclusion in the review.

The selection of studies followed a structured screening process based on predefined inclusion and exclusion criteria. Only original research articles were considered eligible for inclusion. Conference proceedings, book chapters, review papers, and documents with incomplete or inconsistent methodological descriptions were excluded to maintain the scientific rigor of the dataset [43]. The screening process was conducted in accordance with PRISMA guidelines and is summarized in Figure 1. An initial set of 149 records was identified from the database search. After removing 8 non-article documents (e.g., conference abstracts, editorials, review papers), 141 records were subjected to title screening.

Title screening phase: Records were included if they contained (i) explicit references to the target genera (*Asphodelus*, *Asphodeline*) in the title, (ii) references to the Asphodelaceae family in general, or (iii) ambiguous terminology requiring further examination through abstract screening. Records were excluded if they clearly focused on non-target genera or unrelated taxonomic groups. This phase resulted in the exclusion of 56 studies, leaving 85 records for abstract evaluation. Abstract screening phase: Abstracts were assessed to verify thematic alignment with the PICOL framework (Table 2). Records were excluded if they addressed specifically clinical or pharmacological contexts unrelated to any agronomic, ecological, or phytochemical context of research on the target genera. Eight additional records were excluded at this stage, yielding 77 full-text articles for eligibility assessment. Full-text screening phase: Full-text articles were evaluated based on two criteria: (i) accessibility for complete review, and (ii) relevance to the outcomes defined in the PICOL framework. Thirteen studies were excluded due to inaccessibility (*n* = 7), preventing verification of results, or lack of relevance (*n* = 6), where reported findings did not align with the defined research objectives.

In this study, a record was operationally classified as “out-field” if it satisfied at least one of the following criteria: (i) it referred to a non-target taxon retrieved through the wildcard “asphodel*” (e.g., *Anemarrhena asphodeloides*, *Narthecium* spp.); (ii) it addressed clinical, pharmacological, or therapeutic contexts outside the agronomic, ecological, or phytochemical scope defined by the PICOL framework (Table 2); or (iii) it focused on excluded genera (*Aloe*, *Kniphofia*, *Hemerocallis*, *Bulbine*). All out-field records were excluded prior to the computation of bibliometric metrics and network maps.

This systematic procedure resulted in a final dataset of 64 studies (Figure 1). This multi-step screening approach ensured a transparent and reproducible selection process, minimizing bias and enhancing the reliability of the subsequent analysis (Table 3). The complete screening process, including all 149 records initially retrieved from Scopus together with their inclusion/exclusion outcomes, is provided as Supplementary Table S1. The final dataset of the 64 records included in the analysis, exported from Scopus and used as input for all bibliometric computations performed with Bibliometrix and VOSviewer, is provided as Supplementary Table S2.

### 2.4. Descriptive Bibliometric Analysis

Descriptive bibliometric analysis was performed using the Bibliometrix package implemented in RStudio (version 4.5.1), which provides a comprehensive framework for quantitative research assessment and science mapping. The dataset was exported from Scopus in CSV format, including information on authors, titles, publication year, source title, citations, DOI, affiliations, abstracts, keywords, and document type, to ensure a comprehensive and reproducible bibliometric analysis. Descriptive indicators, such as publication output, citation metrics, and authorship patterns, were calculated to characterize the structure and development of the research field. Annual publication trends were analyzed to identify growth patterns and shifts in research interest over time. Collaboration indices, including co-authorship networks and degrees of international collaboration, were derived to assess the extent of scientific cooperation across institutions and countries. These descriptive metrics provided a foundational understanding of the field’s productivity and collaborative landscape before progressing to more complex relational analysis.

### 2.5. Network Analysis and Thematic Mapping

To complement the descriptive analysis, network-based approaches were employed to explore relationships among key variables. Keyword co-occurrence and thematic clustering were analyzed using VOSviewer (version 1.6.20). The dataset was exported from Scopus in CSV format. Before analysis, the dataset was preprocessed to improve data consistency through keyword normalization, including the unification of synonyms, singular and plural forms, and spelling variants, as well as the removal of non-informative terms, e.g., alcohol, allium cepa, anemarrhena asphodeloides, article, narthecium, wild onion. For keyword mapping, co-occurrence analysis was performed using all keywords as the unit of analysis, and the full counting method, with association strength applied as the normalization method. Keywords with a minimum occurrence of three were included in the map. The final keyword co-occurrence network consisted of 50 items distributed across four clusters, with 522 links and a total link strength of 853. This software enables the visualization of complex bibliometric networks, allowing the identification of dominant research themes, conceptual structures, and emerging trends [44]. The integration of statistical indicators with network visualization tools provided a multidimensional perspective on the literature. This approach facilitated not only the quantification of scientific output but also the interpretation of thematic connections and research dynamics over time, offering a holistic view of the intellectual landscape surrounding *Asphodelus* and *Asphodeline* research.

## 3. Results and Discussion

Following the systematic PRISMA-based screening procedure detailed in the Section 2 (Figure 1), a total of 64 studies met the inclusion criteria, corresponding to a 43% inclusion rate. This systematic reduction demonstrates a transparent and reproducible approach, which strengthens the reliability and scientific robustness of the review.

### 3.1. General Aspects of the References Relating to the Research Objectives

**Figure 2:**
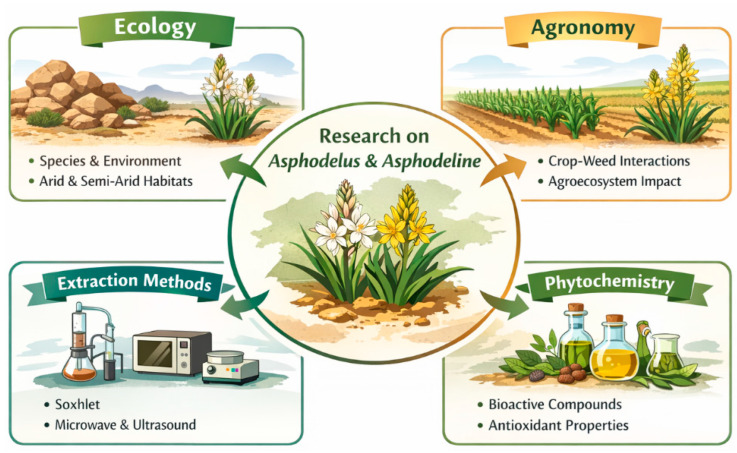
Schematic depiction of the main topics of research identified and analyzed, focusing on species-environment interactions, agroecosystem dynamics, and bioactive compounds and their characterization.

Following the screening and inclusion phases, the final dataset was subjected to a bibliometric analysis using the bibliometrix package, an open-source tool for quantitative research assessment and science mapping that enables analyses of bibliographic data by integrating descriptive statistics, performance indicators, and network-based approaches within a unified analytical framework [45]. The resulting descriptive indicators provide an overview of the structural characteristics of the literature on *Asphodelus* and *Asphodeline*. The dataset comprises 64 documents distributed across 57 sources, indicating that research on these genera is not concentrated within a single disciplinary outlet but rather spans several areas of interest, and this dispersion reflects the interdisciplinary nature of the field (Figure 2).

Among the studies included, those classified in the ecological field, investigating the interaction between geomorphological structures, hydrological regulation, and spatial distribution of *Asphodelus ramosus* L., provide information on the relationships between species and the environment in arid and semiarid conditions [13]. Studies in the agronomic field treat the target species mainly in the context of crop-weed interactions. This approach reflects a prevailing trend in the literature, in which the genera *Asphodelus* and *Asphodeline* are studied mainly as spontaneous or weed components of agroecosystems, with an emphasis on interference dynamics, competitive effects, and management implications, rather than on direct cultivation practices [46]. In the field of extraction and phytochemistry, the work of Eddaoudi et al. systematically compares different extraction methods, including Soxhlet, microwave-assisted, and ultrasound-assisted extraction, for *Asphodelus tenuifolius* Cav. seed oil, evaluating their effects on phytochemical composition, fatty acid and sterol profiles, total phenolic content and antioxidant activity, thus illustrating how the extraction methodology influences both yield and functional chemical characteristics [47]. The research field appears moderately developed and thematically heterogeneous, characterized by strongly collaborative research practices, moderate cross-national collaboration consistent with the geographically constrained distribution of the taxa.

### 3.2. Scientific Production and Citation Trends

#### 3.2.1. Temporal Trends in Scientific Output and Citation

**Figure 3:**
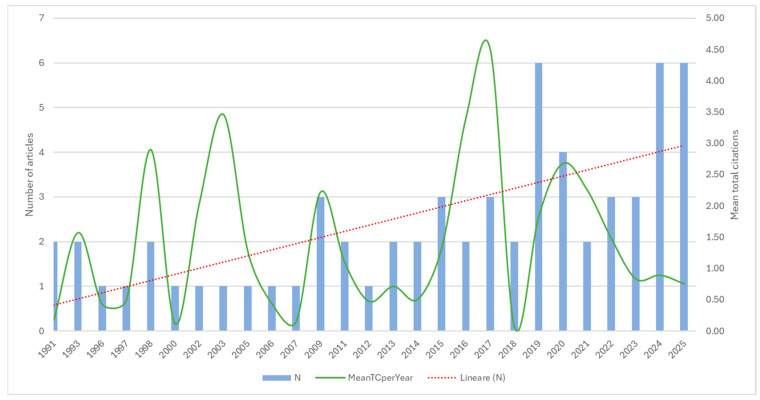
Annual distribution of the publications included, showing the number of articles published per year (N = number of articles), the mean number of citations per year (MeanTCperYear = mean total citations per year), and the linear publication trend over time (Linear (N) = linear trend of the annual number of articles).

Figure 3 shows the temporal evolution of scientific output from 1987 to 2026, with the annual number of publications indicating a gradual and consistent upward trend, as underlined by the linear regression line. Although publication output was sporadic in the early years, it became more sustained over time, with peaks around 2019–2021 and in recent years, indicating growing scholarly interest in the topic. Mean citations per article fluctuate sharply, with peaks in the early 2000s and around 2017, likely driven by a small number of highly cited papers published in years of lower output. This divergence underscores a well-known bibliometric pattern between publication growth and citation impact. While scientific productivity has increased steadily, citation impact has not risen proportionally, likely due to citation latency in recent publications and the dilution effect of higher output volumes.

#### 3.2.2. Citation Impact in the Literature

Table 4, reporting the most globally cited documents, provides additional evidence for the citation dynamics previously discussed. As already observed in the temporal analysis of publication output and mean citation rates, the impact of citations is unevenly distributed across the literature, being largely concentrated in a limited number of publications. Many of these publications were released between the early 1990s and the mid-2000s. These documents continue to account for a substantial proportion of total citations, despite the subsequent increase in publication volume [48]. Several of the most cited contributions were published in high-impact journals such as Oecologia, New Phytologist, Industrial Crops and Products, and Journal of Agricultural and Food Chemistry. These outlets typically host studies addressing ecological processes, plant functional traits, or biochemical properties with relevance beyond a single taxon, which should be explicitly acknowledged as a structural feature of citation-based bibliometric analyses and partly explains their sustained citation performance over time, even when the target genera are not the exclusive focus of those studies. More recent highly cited papers are primarily associated with phytochemical research [49]. Despite their limited number, these studies have received relatively high citation counts, suggesting that phytochemical-focused research can gain strong visibility when linked to widely studied biological targets.

Table 4 distinguishes total citations from normalized impact. Older papers usually have more citations because they have been available longer, whereas Normalized TC reflects performance relative to similar studies. Thus, some recent papers show high normalized impact despite fewer citations, while some highly cited papers perform only in line with expectations for their context.

#### 3.2.3. Country-Level Citation Network

**Figure 4:**
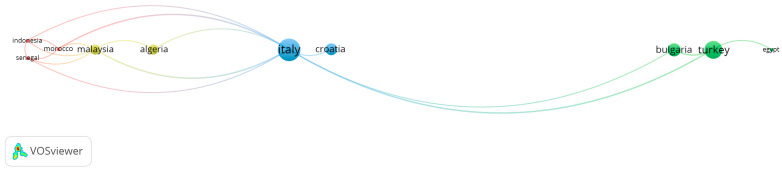
Country citation network generated with VOSviewer; node size indicates citation impact, and links represent inter-country citation relationships.

The spatial distribution of citation networks related to the genera *Asphodelus* and *Asphodeline* was explored through a country-level bibliometric mapping performed using VOSviewer, a widely adopted software for constructing and visualizing bibliometric networks based on co-authorship, citation, and co-occurrence data [50]. While several countries contribute a comparable number of documents, the citation-based network (Figure 4) reveals notable differences in impact.

Some productive countries appear in relatively peripheral positions in the citation map, which may indicate that publication volume does not necessarily translate into comparable citation impact. Turkey appears to maintain visibility in both the production and citation networks, suggesting a possible balance between output and recognition. Bulgaria and Egypt, despite a lower publication volume, are also represented in the citation network, which may reflect the influence of a limited number of cited contributions. Italy occupies a central position in the collaboration network and shows the largest node and the highest number of connections, suggesting a potentially important bridging role between different geographic areas. Collectively, the network structure appears closer to a radial configuration than to a polycentric one.

### 3.3. Core Journals and Source Distribution

**Figure 5:**
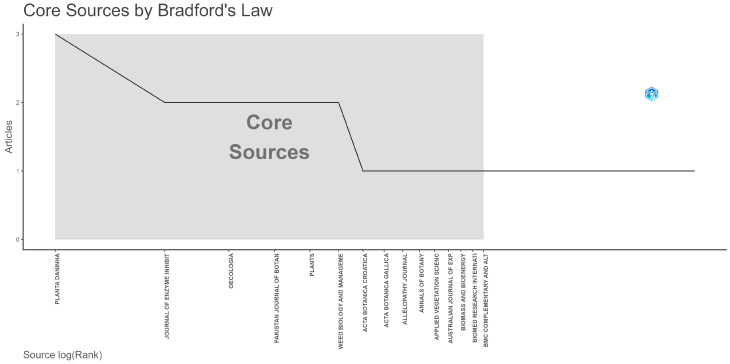
Bradford’s Law plot showing journals by rank and number of articles, with the shaded area indicating the core sources.

Figure 5 illustrates the identification of core journals according to Bradford’s Law, showing how publications on *Asphodelus* and *Asphodeline* are unevenly distributed across sources. As predicted by Bradford’s model, a small set of journals constitutes the core of the literature, while a larger number of outlets contribute only sporadically [51]. Journals located in the core zone reflect the breadth of the subject rather than specialization around a single research focus. For example, *Planta Daninha*, *Pakistan Journal of Botany,* and similar agronomy-oriented journals often publish studies in which *Asphodelus* species are treated in relation to cropping systems, often as weeds or interferers [52,53]. Other journals, including Plants, are more focused on studies concerning extraction techniques, yields, and phytochemical profiles of the extracts, with attention to potential agronomic applications [54]. Journals ranked subsequently in the Bradford classification provide fewer articles but remain scientifically relevant due to their thematic specificity. Studies published in multidisciplinary or natural product-oriented journals often focus on the chemical composition and potential of resources, as illustrated by investigations into wild oil plants that include *Asphodelus* species among unconventional oil sources [55]. These contributions highlight an applied phytochemical perspective that complements, rather than replaces, ecological and agronomic research.

### 3.4. Geographical Distribution of Scientific Production

**Figure 6:**
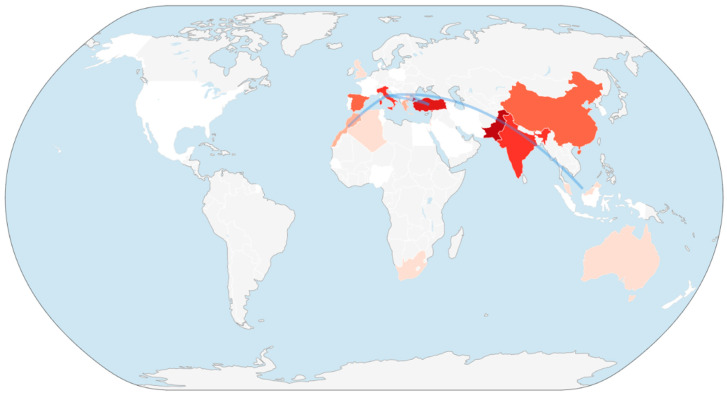
Global geographic distribution of country-level collaboration intensity; darker shading indicates higher research output, while arcs represent major international collaboration flows.

The geographical representation of the globe extracted through data analysis (Figure 6) clearly and visually indicates that scientific research on *Asphodelus* and *Asphodeline* is strongly concentrated in a specific geographical area, rather than being evenly distributed worldwide. Italy emerges as one of the most cited countries and the main hub of international collaboration, showing the highest level of connectivity with other countries. In contrast, Turkey and Pakistan appear as the most productive countries in terms of scientific output. Co-authorship links are predominantly aligned along a Mediterranean–South Asian axis, consistent with the natural distribution range of the studied genera, suggesting that research activity tends to cluster in regions where the species actually occur. Contributions from the Americas and Northern Europe are almost absent, suggesting that research on those genera remains closely linked to local ecological conditions and regional research contexts, rather than reflecting truly global research effort.

The map shows contributions from countries located beyond the core Mediterranean area, including Turkey and Egypt, which appear as distinct but connected nodes. Turkey emerges as a relevant contributor. The spatial arrangement of nodes suggests that document production follows a biogeographical logic, where countries hosting natural populations of the target genera are also generating most of the scientific output. Italy occupies a central position in the collaboration network, despite a relatively modest document output, further reinforcing the geographically coherent structure of a research landscape concentrated within the Mediterranean Basin and adjacent regions. Southern Mediterranean countries, such as Morocco and Algeria, are clearly represented, indicating consistent scientific activity in areas where *Asphodelus* and *Asphodeline* naturally occur and are traditionally utilized. Scientific production has expanded primarily in countries within the natural distribution range of *Asphodelus* and *Asphodeline*, reflecting ecological availability and local research priorities. Over time, this production has been accompanied by a consolidation of citation impact in a smaller subset of countries, particularly those contributing integrative, methodologically robust, or conceptually influential studies.

### 3.5. Keyword Co-Occurrence and Thematic Cluster Analysis

The keyword co-occurrence analysis displays the most frequently occurring keywords extracted from the analyzed literature. These keywords offer valuable insight into the conceptual structure of the field, highlighting recurring themes that define the knowledge base surrounding these studies.

**Figure 7:**
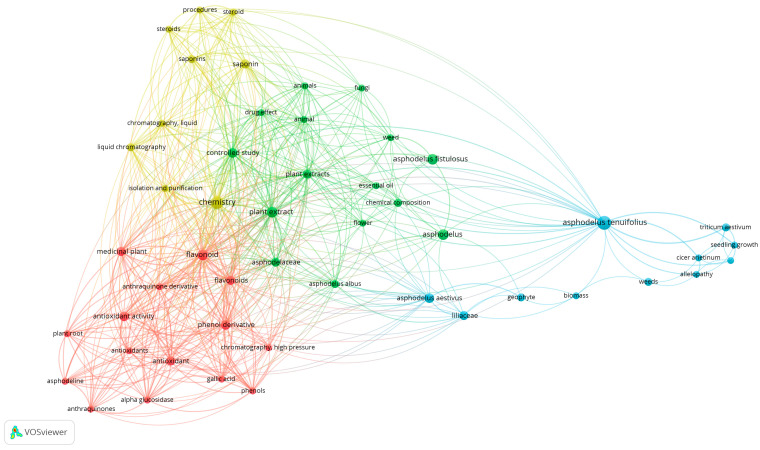
Keyword co-occurrence network map generated with VOSviewer, displaying thematic clusters in different colors.

Figure 7 reveals a structured thematic organization of the literature articulated into four major clusters, clearly distinguishable and strongly interconnected. This structure becomes analytically valuable when interpreted through network indicators such as the number of connections, the number of occurrences, and the total strength of the connections, which together enable the identification of thematic relevance, relational density, and integrative capacity within the field.

#### 3.5.1. Blue Cluster: Ecology and Agronomy

The blue cluster, primarily associated with ecological and agronomic aspects, is organized around *Asphodelus tenuifolius* Cav., which exhibits 30 links, 12 occurrences, and a total link strength of 46. The 12 occurrences, the highest frequency among the reported terms, emphasize the taxonomic centrality of the species within the corpus. However, the comparatively lower total link strength suggests that, while frequently mentioned, the species name does not generate the same degree of integrative connectivity as thematic constructs such as chemistry or flavonoids. In other words, the main point of the argument seems to be species-focused on frequency but concept-driven in structural cohesion. A similar pattern can be observed for *Asphodelus aestivus* Brot. which shows 23 links, 6 occurrences, a total link strength of 25, and related taxa in green adjacent cluster: *Asphodelus* displays 14 links, 7 occurrences, and a total link strength of 15; *Asphodelus fistulosus* Cav. records 6 links, 7 occurrences, and a total link strength of 8. These comparatively lower values may indicate a more localized or peripheral integration within the network.

#### 3.5.2. Green Cluster: Plant Extract

The green cluster performs a transversal methodological role. Plant extract, with 39 links, 7 occurrences, and a total link strength of 74, occupies a strategic position, functioning as an operational bridge between raw plant material and both compositional and functional studies. Controlled study, with 34 links, 6 occurrences, and a total link strength of 66, shows a level of connectivity that is comparable to that of core phytochemical and bioactivity terms. This suggests that standardized experimental frameworks are deeply incorporated into the research architecture. The relatively high number of links indicates frequent integration with both chemical and biological keywords, while the total link strength shows that these associations are recurrent and not incidental.

#### 3.5.3. Yellow Cluster: Phytochemical Investigation and Analytical Characterization

The yellow cluster centers on phytochemical investigation and analytical characterization. Chemistry records 40 links, 10 occurrences, and a total link strength of 111, the highest value among the considered keywords. This quantitative prominence advises that chemical characterization permeates virtually all thematic domains, acting as the core of the network. The high total link strength reflects a dense web of co-occurrences, implying that discussions of composition systematically intersect with bioactivity testing, extraction procedures, and methodological validation. The presence of saponin, characterized by 25 links, 5 occurrences, and a total link strength of 50, recommends that specific metabolite classes contribute meaningfully to the cohesion of this cluster. Collectively, these indicators contribute to the definition that phytochemical analysis is not ancillary, but structurally central to the organization of knowledge in this field.

#### 3.5.4. Red Cluster: Bioactivity

The red cluster exhibits the strongest structural integration, focusing on bioactivity and phenolic constituents, structured around flavonoid, which display 38 links, 7 occurrences, and a total link strength of 85. These values indicate not only a consistent presence in the corpus, but above all a high degree of co-occurrence with other terms. In network terms, this means that flavonoids function as a relational hub connecting phytochemical analysis to biological evaluation. Antioxidant activity, with 25 links, 6 occurrences, and a total link strength of 54, shows a slightly lower but still robust connectivity profile, suggesting that antioxidant assessment represents one of the most consolidated and standardized bioassays in the literature. The term medicinal plant, characterized by 32 links, 5 occurrences, and a total link strength of 59, further reinforces the applied and phytochemical framing of this thematic area. The convergence of these links is an indication that bioactivity constitutes a consistent and well-established research axis, with significant developments in these directions.

Considering the evidence presented, the integrated interpretation of clusters and keyword co-occurrences delineates a hierarchically structured intellectual framework. Within this structure, chemistry emerges as the principal organizing axis. Terms such as flavonoids and plant extracts function as key integrative nodes, linking chemical characterization with biological applications, while the term-controlled study contributes to methodological standardization and consolidation. In contrast, *Asphodelus tenuifolius* Cav., despite its recurrence within the corpus, exerts a comparatively limited integrative influence, indicating that thematic cohesion is driven predominantly by analytical and bioactivity-oriented research frameworks.

### 3.6. Keyword Co-Occurrence Correlated with Its Temporal Evolution

**Figure 8:**
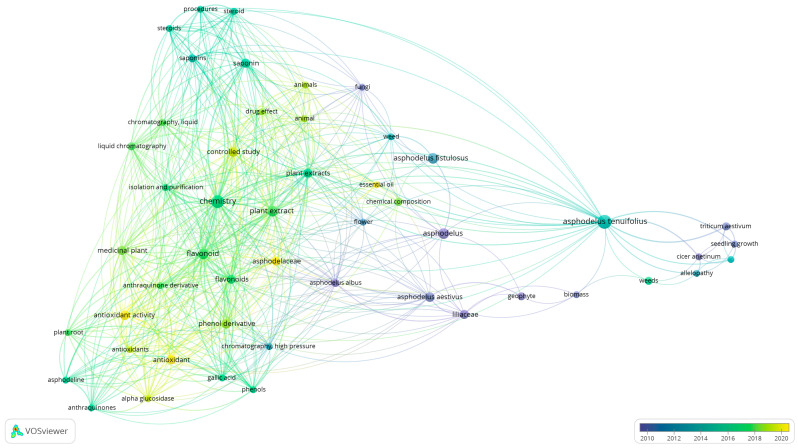
Keyword co-occurrence network visualized with VOSviewer. The map highlights major themes, with node colors indicating the average publication year. Stronger linkages represent higher co-occurrence frequency.

A temporal dimension is incorporated into the bibliometric analysis, illustrating the evolution of the main research topics over time (Figure 8). The overlay visualization reveals not merely a chronological succession of keywords, but a progressive refinement in the way the genera *Asphodelus* and *Asphodeline* have been investigated, moving from descriptive foundations to chemical and biological integrated frameworks and to ecological contextualized applications.

In the earlier phase of the literature, the emphasis appears anchored in taxonomic positioning and primary compositional insights, establishing the botanical identity and chemical potential of the taxa under study. Within this initial trajectory, the genera are predominantly framed within an ecological–agronomic context, where their biological relevance is defined by interactions with cropping systems and their role as weeds [56,57]. Studies on crop interference and allelopathic effects emphasize their impact on plant growth and development, while additional investigations further contextualize their persistence and adaptive traits within agroecosystems, reinforcing persistence and adaptive plasticity as a geophytic weed [58].

As the literature evolves, the focus shifts from ecological description to laboratory-based chemical characterization, emphasizing compositional specificity and analytical standardization. Essential oil profiling in *Asphodelus aestivus* Brot. highlights organ-specific chemical fingerprints and associated bioactivities [59], while studies on *Asphodelus tenuifolius* Cav. demonstrate optimized extraction processes and well-defined chemical compositions [47]. Parallel investigations on *Asphodeline* and comparative analyses of *Asphodelus tenuifolius* Cav. extracts further integrate chemotaxonomic, biochemical, and bioactivity assessments, reflecting a mature and analytically driven research phase [60,61]. The most recent stage within the temporal gradient represented in the visualization is marked by a pronounced integration of compositional analysis with functional validation, particularly through the investigation of phenolic compounds and antioxidant-related bioactivity. In this context, LC–MS/MS-based characterization of phenolic constituents in *Asphodelus aestivus* Brot. is directly correlated with strong bioactivities [62].

Taken together, the chronological sequence evidenced in the overlay visualization suggests a coherent progression: from early ecological and weed-science investigations, through increasingly rigorous phytochemical and extraction methodologies, to analytically resolved and biologically validated frameworks in which chemical profiling, functional assays, and taxonomic depth converge. This trend reflects that the scientific approach to *Asphodelus* and *Asphodeline* has evolved from foundational botanical characterization toward structurally integrated and experimentally substantiated domains of inquiry.

### 3.7. Identification of Driving Thematics

**Figure 9:**
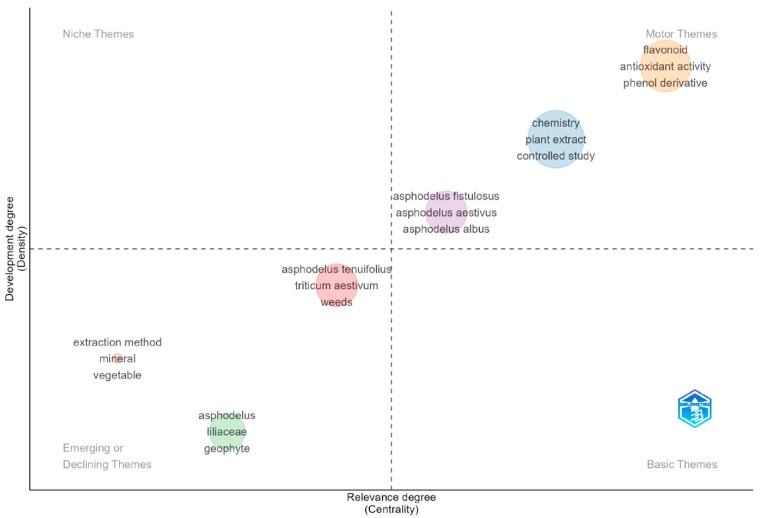
Thematic map showing the structural position of research topics. Themes are distributed into four quadrants: motor themes, niche themes, emerging or declining themes, and basic themes, according to their degree of relevance (centrality, x-axis) and level of development (density, y-axis).

As a final addition to what has been explored up to this point, the thematic map displays the results of the analysis (Figure 9), mapping the research topics on two dimensions: degree of relevance (centrality) on the x-axis and density on the y-axis. The diagram is divided into four quadrants by dashed lines. The Motor Themes quadrant in the upper right contains well-developed and central themes, including flavonoid, antioxidant activity, phenol derivative, chemistry, plant extract, and controlled study. Furthermore, authors such as Kitouni et al. [63] and Polat Köse [62], whose recent research focuses precisely on these topics, show exactly what is highlighted in the thematic map. This observation represents the core focus and actual leading theme of the literature with high relevance and extensive research, due to the phytochemical aspects and extraction methods of plant-derived bioactive compounds. These themes reflect the primary research interest in characterizing the chemical composition and therapeutic potential of natural products, particularly their antioxidant properties and phenolic content, as reported from Eddine et al. [61], which are evaluated through rigorous controlled study designs and in vitro experiments.

A closer examination of the literature indicates that the centrality of these themes is primarily driven by advances in analytical and bioassay methodologies. Techniques such as LC–MS/MS have enabled precise identification and quantification of bioactive compounds, particularly phenolics like chlorogenic acid, directly linked to strong antioxidant and therapeutic potential [64]. In parallel, combined in vitro and in silico approaches have demonstrated that flavonoid derivatives, especially luteolin-based compounds, exert significant inhibitory effects on enzymes involved in metabolic disorders, reinforcing their pharmacological relevance [65]. This integration of chemical profiling and functional validation reflects a broader shift toward structure–activity relationship–oriented research.

The importance of phenolics and flavonoids is further confirmed by studies on *Asphodelus* spp., which report pronounced antioxidant, enzyme inhibitory, and antimicrobial properties, including antibiofilm activity [66]. Such evidence highlights a transition from purely compositional analyses to application-driven research, particularly in addressing microbial resistance. Similarly, investigations on essential oils reveal considerable chemical variability alongside notable antibacterial effects, expanding the functional scope of plant-derived metabolites [67]. This phytochemical framework is complemented by research on related taxa such as *Asphodeline*, where the concept of “multicomponent patterns” emphasizes synergistic interactions among secondary metabolites (phenolics, flavonoids, anthraquinones) [50]. In this context, robust analytical tools, such as UHPLC for anthraquinone quantification, are essential to ensure reproducibility and standardization [68]. Meanwhile, a wide range of biological activities (antioxidant, anti-inflammatory, enzyme inhibitory, antimicrobial) has been consistently documented [69,70,71].

Themes located in the emerging or declining quadrant, rather than indicating a true decline, may reflect a transition toward more integrative and application-oriented research frameworks, where traditional taxonomic and methodological topics are embedded within broader phytochemical and pharmacological studies. An intermediate position is occupied by specific *Asphodelus* species (*A. fistulosus, A. aestivus, A. albus, A. tenuifolius*), probably associated both with agronomic concerns due to their role as weeds in crops such as *Triticum aestivum* [53] and with increasing interest in their bioactive properties exhibiting strong antioxidant and antifungal activities along with potential practical applications in dermocosmetics through the inhibition of collagenase and tyrosinase [72].

## 4. Conclusions

This bibliometric analysis provides a structured interpretation of how research on *Asphodelus* and *Asphodeline* has evolved over the past four decades, revealing not only quantitative growth but also a clear qualitative reconfiguration of the field. Rather than representing a uniformly expanding domain, the literature has progressively shifted toward phytochemical characterization and bioactivity-driven investigations, while agronomic and domestication-oriented studies remain comparatively underdeveloped.

This imbalance has important implications. The strong emphasis on analytical and bioassay methodologies has enabled significant advances in the identification and functional validation of bioactive compounds. However, the limited integration with agronomic research continues to constrain the practical exploitation and large-scale valorisation of these species. In this sense, the current research landscape can be interpreted as methodologically mature yet still fragmented in terms of application. The results also suggest that thematic evolution is not simply linear but reflects a progressive convergence between chemical profiling, biological validation, and emerging application domains, including nutraceutical, pharmaceutical, dermo-cosmetic, and agroecological contexts. This convergence underscores the need for more integrative research frameworks capable of linking phytochemical potential with cultivation strategies, sustainability assessment, and value-chain development. In this perspective, future research should prioritize the development of standardized cultivation and propagation protocols, the integration of phytochemical data with agronomic performance and environmental adaptability, and the exploration of translational pathways that connect laboratory-scale bioactivity with industrial and commercial applications across sectors such as functional foods, natural health products, and bio-based formulations. Furthermore, expanding the geographical and disciplinary scope of research may help reduce the current regional concentration and enhance the global relevance of these taxa. Overall, this study does not merely map existing knowledge but identifies a critical transition point: advancing from predominantly descriptive and analytical approaches toward integrated, application-oriented systems will be essential to fully unlock the potential of these still underexplored Mediterranean plant resources.

## Figures and Tables

**Table 1:** Sensitivity analysis of the Scopus search string. Records retrieved at three progressively restrictive query steps (A: taxa only; B: taxa + intervention + outcomes; C: full final query with NOT clause). Reductions are reported as absolute values, as percentages relative to the previous step, and as proportions of the taxonomy-only baseline.

Search Step	Query Components	Records	Reduction vs. Previous Step	% of Baseline
A	Taxonomic terms only	1797	—	100.0%
B	A + intervention block + outcomes block	452	−1345	25.2%
C	B + NOT (exclusion block)—final query	149	−303	8.3%

**Table 2:** The definition of the terms ‘population’, ‘intervention’, ‘comparison’, ‘outcomes’, and ‘location’ (PICOL).

P	Population	Crops: Asphodelaceae, *Asphodelus ramosus* L., *Asphodelus albus* auct., *Asphodelus fistulosus* L., *Asphodeline lutea* (L.) Rchb., *Asphodeline liburnica* (Scop.) Rchb., *Asphodelus liburnicus* Scop., *Asphodeline taurica* (Pall.) Endl., *Asphodelus delphinensis* Gren. & Godr., *Asphodelus pyrenaicus* Jord., *Asphodelus luteus* L., *Branched asphodel*, Mediterranean asphodel, yellow asphodel Excluded: Aloe, *Aloe Vera*, kniphofia, Hemerocallis, day lily, bulbine
I	Intervention	Cultivation, agronomic management, propagation, seed germination, micropropagation, in vitro culture, soil management, organic soil improvers, fertilization, abiotic stress, biotic stress, genetic selection, domestication, extraction, metabolite extraction, post-harvest processing
C	Comparison	Different cultivation practices, different propagation or extraction methods,
O	Outcomes	Biomass, yield, extract, metabolite profile, flavonoid content, antioxidant activity
L	Location	Globe

**Table 3:** Inclusion and exclusion criteria applied at the title, abstract, and full-text screening stages for the selection of studies, according to taxonomic relevance and consistency with the PICOL framework.

Screening Phase	Inclusion Criteria	Exclusion Criteria
Title	Explicit mention of *Asphodelus* or *Asphodeline* Reference to the Asphodelaceae family Ambiguous content requiring abstract review	Focus on non-target genera Clearly unrelated taxonomic groups
Abstract	Alignment with PICOL framework Agronomic, ecological, or phytochemical focus	Clinical/pharmacological contexts unrelated to target genera Insufficient thematic relevance
Full text	Accessible for complete review Results aligned with PICOL outcomes	Inaccessible full text Results not matching defined research objectives

**Table 4:** Top cited articles included, ranked by total citations (Total Citations = overall number of citations received by each article) and normalized citation impact (Normalized TC = normalized total citations), with corresponding journal source and Digital Object Identifier (DOI).

Articles	Journal	DOI	Total Citations	Normalized TC
Pascual-Villalobos, 1998	Ind. Crop. Prod.	10.1016/S0926-6690(98)00002-8	168	2.00
Tuberoso, 2009	J. Agric. Food Chem.	10.1021/jf803991j	87	2.17
Cavagnaro, 2003	New Phytol.	10.1046/j.1469-8137.2003.00654.x	83	1.00
Obeso, 1993	Oecologia	10.1007/BF00328967	78	1.46
Locatelli, 2017	J. Enzym. Inhib. Med. Chem.	10.1080/14756366.2016.1235041	70	1.56
Zengin, 2016	J. Enzym. Inhib. Med. Chem.	10.3109/14756366.2015.1063623	68	1.81
Salhi, 2017	Biomed Res. Int.	10.1155/2017/7526291	61	1.36
Ali, 2020	Biomass Bioenergy	10.1016/j.biombioe.2020.105595	54	2.88
Mehmood, 2019	Plant Physiol. Biochem.	10.1016/j.plaphy.2018.11.029	51	3.48
Somerville, 2002	Aust. J. Exp. Agric.	10.1071/EA01086	51	1.00

## Data Availability

The original contributions presented in this study are included in the article/Supplementary Materials. Further inquiries can be directed to the corresponding author.

## Supplementary Materials

The following supporting information can be downloaded at https://www.mdpi.com/article/10.3390/plants15101421/s1.
